# Construction of Core–Shell MOF@COF Hybrids with Controllable Morphology Adjustment of COF Shell as a Novel Platform for Photocatalytic Cascade Reactions

**DOI:** 10.1002/advs.202101884

**Published:** 2021-08-10

**Authors:** Meng‐Yao Zhang, Jun‐Kang Li, Rui Wang, Shu‐Na Zhao, Shuang‐Quan Zang, Thomas C. W. Mak

**Affiliations:** ^1^ Henan Key Laboratory of Crystalline Molecular Functional Materials Henan International Joint Laboratory of Tumor Theranostical Cluster Materials Green Catalysis Center and College of Chemistry Zhengzhou University Zhengzhou 450001 P. R. China

**Keywords:** core–shell structure, MOF@COF hybrids, morphology adjustment, photocatalytic cascade reactions, photo‐generated carriers’ separation

## Abstract

Recently, novel core–shell MOF@COF hybrids display excellent performance in various fields because of their inherited advantages from their parent MOFs and/or COFs. However, it is still a grand challenge to adjust the morphology of MOFs and/or COFs for consequent performance improvement. Herein, a Ti‐MOF@TpTt hybrid coated with ultra‐thin COF nanobelt, which is different from the fibrillar‐like parent COF, is successfully synthesized through a sequential growth strategy. The as‐obtained Pd decorated Ti‐MOF@TpTt catalyst exhibits much higher photocatalytic performance than those of Ti‐MOF, TpTt‐COF, and Ti‐MOF@TpTt hybrids with fibrillar‐like COF shell for the photocatalytic cascade reactions of ammonia borane (AB) hydrolysis and nitroarenes hydrogenation. These can be attributed to its high BET surface area, core–shell structure, and type II heterojunction, which offers more accessible active sites and improves the separation efficiency of photo‐generated carriers. Finally, the possible mechanisms of the cascade reaction are also proposed to well explain the improved performance of this photocatalytic system. This work presents a constructive route for designing core–shell MOF@COF hybrids with controllable morphology adjustment of COF shell, leading to the improved photocatalytic ability to broaden the applications of MOF/COF hybrid materials.

## Graphical abstract

A novel Ti‐MOF@TpTt hybrid, which coats with ultrathin TpTt nanobelt as the shell, is successfully synthesized through a sequential growth strategy. The as‐obtained Pd decorated Pd@Ti‐MOF@TpTt catalyst exhibits much higher photocatalytic performance than Ti‐MOF, TpTt‐COF, and the hybrids with fibrillar‐like COF‐shell for the cascade reactions of AB hydrolysis and nitroarenes hydrogenation.

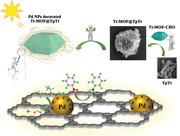

## 1 Introduction

The current issues of the global energy crisis and environmental problems, which are caused by the combustion of fossil fuels, have promoted the exploration of sustainable and clean energy alternatives.^[^ ^1^ ^]^ Solar energy is one of the most promising candidates to replace fossil fuels because of its advantages of eco‐friendly, inexpensive, and inexhaustible.^[^ ^2^ ^]^ Photocatalysis has been a promising technology to convert abundant solar energy into chemical energy, which has received enormous scientific attention.^[^ ^3^ ^]^ Over the past decades, various materials have been developed for photocatalysis, including transition metal complexes, metal oxides, metal sulfides, and graphitic carbon nitride, etc.^[^ ^4^ ^]^ However, the efficiency of photocatalysis is still far from satisfactory for practical application, mainly owing to the severe recombination of photo‐generated electrons and holes.^[^ ^5^ ^]^ Thus, designing new photocatalysts with excellent carrier separation efficiency is still a big challenge for the commercialization of photocatalysis technology.

Covalent organic frameworks (COFs) are an emerging class of crystalline porous materials, formed by linking organic building units into predictable structures through strong covalent bonds.^[^ ^6^ ^]^ Since the pioneering work by Yaghi in 2005,^[^ ^7^ ^]^ COFs have flourished due to their characteristic features, such as low density, regular pore structure, large specific surface area, and tunable function, which endow them great application potential in gas adsorption and separation, chemical sensing, heterogeneous catalysis, proton conduction, and drug delivery.^[^ ^8^ ^]^ Moreover, COFs have been proven to be an excellent photocatalyst for various photocatalytic reactions, including water splitting, CO_2_ reduction, pollution degradation, and organic transformations, due to the following reasons: i) the abundant organic building units provide great opportunities for COFs with tunable bandgaps; ii) the extensive п‐conjugated skeleton in COFs can ensure the mobility of photo‐generated carriers; iii) the large specific surface area of COFs enables the reactant transportation.^[^ ^9^ ^]^ Although most of COFs exhibit relatively low photocatalytic efficiency due to the rapid recombination of photo‐generated electron‐hole pairs, several strategies have been developed for improving the separation and migration efficiency of photo‐generated carriers in COF photocatalysts. For example, an ultrathin 2D COF nanosheet synthesized through an imine‐exchange strategy shows excellent photocatalytic performance for CO_2_‐to‐CO conversion far superior to corresponding bulk materials due to abundant accessible active sites on the surface.^[^ ^10^ ^]^ Besides, several photoactive COF‐based hybrids, such as COFs/metal oxides, COFs/molecular catalysts, COFs/Mxene, and COFs/perovskite were reported, exhibiting improving photocatalytic activity on account of efficient photo‐generated carriers’ separation and transfer between COFs and the other components.^[^ ^11^ ^]^ More recently, novel core–shell MOF@COF hybrids were achieved by introducing the as‐prepared MOFs into the synthetic process of COFs.^[^ ^12^ ^]^ Such core–shell MOF@COF hybrids inherit the high surface area of the parent porous MOF and COF materials. The spatial separation and the strong covalent connection between MOF and COF effectively facilitate the charge transfer between two components, endowing core–shell MOF@COF hybrids with excellent photocatalytic activities for water splitting and degradation of organic dyes.^[^ ^13^ ^]^ However, the synthetic methodology for MOF/COF hybrids always results in the simple combination of MOF and COF in a core–shell structure. It is still a grand challenge to adjust the morphology of MOFs and/or COFs of core–shell MOF@COF hybrids for consequent performance improvement.

In this work, a novel MOF@COF hybrid with ultra‐thin COF nanobelt as the shell, which is different from the fibrillar‐like parent COF, was successfully synthesized through a sequential growth strategy. MIL‐125‐NH_2_(Ti), named Ti‐MOF hereafter, was selected as the MOF core due to its well‐known light‐harvesting ability, large surface area, high thermal and chemical stability, as well as the amino‐functional group for further covalent modifications.^[^ ^14^ ^]^ As for the choice of COF shell, a triazine and keto functionalized TpTt‐COF came into our sight because of its high chemical stability, significant light absorption, and appropriate band edge positions with Ti‐MOF for migrating the photo‐generated electrons to the TpTt shell and leaving the photo‐generated holes in Ti‐MOF.^[^ ^15^ ^]^ In the sequential growth strategy, Ti‐MOF was first reacted with 2,4,6‐triformylphloroglucinol (Tp) to form Ti‐MOF‐CHO with enhanced covalent connecting sites for the TpTt shell growth. Then, the TpTt shell with ultra‐thin nanobelt structure, which afforded more accessible active sites, was realized by rationally controlling the ratio of Ti‐MOF‐CHO and the TpTt‐COF precursors. Pd nanoparticles (NPs) were decorated on the TpTt shell to concentrate the photo‐generated electrons. The as‐obtained Pd decorated Ti‐MOF@TpTt catalysts exhibited much higher photocatalytic performance than Ti‐MOF, TpTt‐COF, and Ti‐MOF@TpTt hybrids with fibrillar‐like COF shell for the cascade reactions of ammonia borane (AB) hydrolysis and nitroarenes hydrogenation. These can be attributed to its high BET surface area, core–shell structure and type II heterojunction, which offers more accessible active sites and improves the separation efficiency of photo‐generated carriers. The possible mechanisms of the improved photocatalytic performance were also explored by ^11^B NMR, kinetic isotopic experiments, and GC‐MS, etc.

## 2 Results and Discussion

**Scheme 1:**
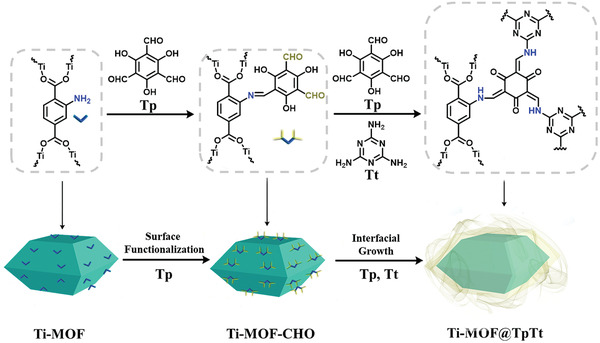
Schematic illustration of the synthesis of Pd decorated Ti‐MOF@TpTt hybrids.

**Figure 1:**
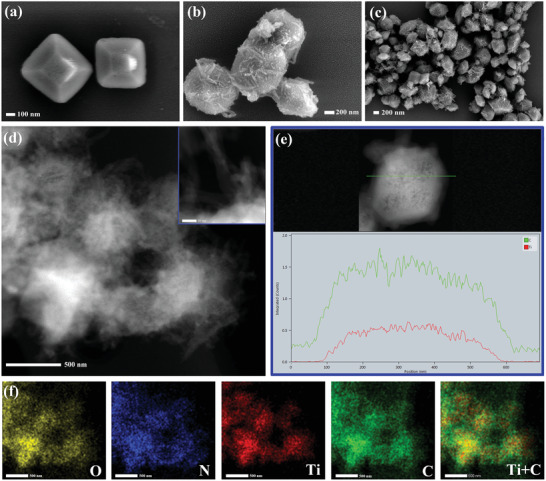
The SEM images of a) Ti‐MOF, b,c) The core–shell structure of **2**; d) The HAADF‐STEM image of **2**. The inset is the enlarged image of **2**; e) EDX line profile of **2**; f) Elemental mapping images of **2**.

The synthetic protocol of Ti‐MOF@TpTt hybrids is depicted in **Scheme** **1**. As the first step, Ti‐MOF was prepared through a typical solvothermal method and showed similar Powder X‐ray diffraction (PXRD) patterns to the previously reported results (Figure S1, Supporting Information),^[^ ^16^ ^]^ suggesting the successful synthesis of Ti‐MOF with high quality. The scanning electron microscopy (SEM) images reveal their morphology of truncated bipyramid octahedron with smooth surfaces (**Figure** **1**a and Figure S2a, Supporting Information). Subsequently, the Ti‐MOF was functionalized with Tp aldehyde through the Schiff base reaction, yielding aldehyde‐modified Ti‐MOF‐CHO. It still maintains the crystalline integrity and similar morphology to Ti‐MOF, which is confirmed by the results of PXRD and SEM (Figures S1 and S2b, Supporting Information). The successful modification of aldehyde groups on Ti‐MOF was further verified by the typical aldehydic C═O stretching vibration at ≈1700 cm^–1^ in the Fourier transform infrared (FTIR) spectra (Figure S3, Supporting Information). The dense aldehyde groups grafted on the surface of Ti‐MOF is very beneficial to the preparation of Ti‐MOF@TpTt hybrids because it could provide more reacting sites for TpTt‐COF growth. Finally, the Ti‐MOF@TpTt hybrids (referred to as **1**–**4** respectively) have been successfully obtained by condensing Tt, Tp, and Ti‐MOF‐CHO via the same synthetic procedure of TpTt‐COF.^[^ ^15^ ^]^ SEM observation reveals that the Ti‐MOF@TpTt hybrids almost inherit the truncated bipyramid octahedron of Ti‐MOF after the surface functionalization (Figure 1b,c). Noteworthy, TpTt‐COF grows vertically on the surface of Ti‐MOF with an ultra‐thin nanobelt structure (Figure 1d inset and S4, Supporting Information), forming a loose shell layer. The high‐angle annular dark‐field scanning transmission electron microscopy (HADDF‐STEM) and EDX‐mapping images clearly confirm the core–shell structure of Ti‐MOF@TpTt hybrids (Figure 1d–f). With an increased amount of TpTt‐COF precursors, TpTt‐COF gradually agglomerates to form a fibrillar‐like structure and tightly wraps Ti‐MOF cores, and then grows in isolation (Figure S5c,d: Supporting Information). This is presumably because the post‐modified aldehyde groups on the surface of Ti‐MOF‐CHO are deficient to confine the growth of TpTt‐COF on Ti‐MOF. In order to further prove the importance of the post‐modified aldehyde groups on the surface of Ti‐MOF, Ti‐MOF was directly used for the synthesis of Ti‐MOF@TpTt (without ‐CHO modification) hybrids. The SEM images of Ti‐MOF@TpTt (without ‐CHO modification) hybrids show that the TpTt shell grows sparsely and unevenly with a low concentration of TpTt‐COF precursors (Figure S6a,b: Supporting Information). When the concentration of TpTt‐COF precursors is high, the morphologies are similar to the Ti‐MOF@TpTt hybrids with aldehyde modification. This is mainly due to the insufficient reacting sites on the Ti‐MOF surface. Thus, the amount of reacting sites on the surface of Ti‐MOF has a significant effect on controlling the morphology of COF shell in core–shell MOF@COF hybrids.

**Figure 2:**
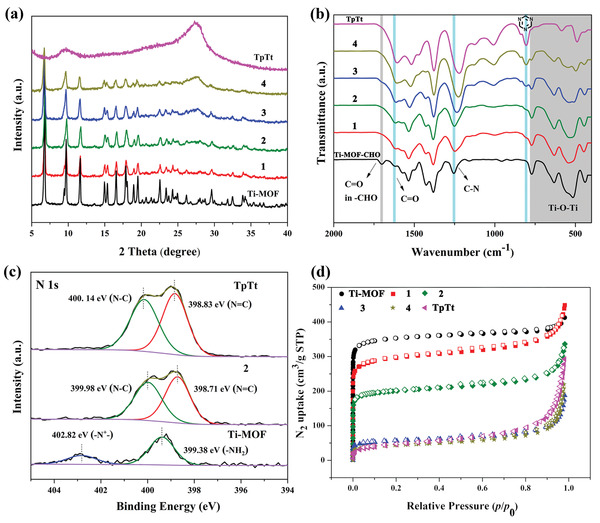
a) The PXRD patterns of Ti‐MOF, TpTt‐COF, and Ti‐MOF@TpTt hybrids; b) FTIR spectra of Ti‐MOF‐CHO, TpTt‐COF, and Ti‐MOF@TpTt hybrids; c) High‐resolution N 1s XPS spectra of Ti‐MOF, **2**, and TpTt‐COF; d) N_2_ adsorption‐desorption isotherms of Ti‐MOF, TpTt‐COF, and Ti‐MOF@TpTt hybrids.

Further structural information of Ti‐MOF@TpTt hybrids was characterized using PXRD, FTIR, and X‐ray photoelectron spectroscopy (XPS), etc. Firstly, the crystalline structure of four Ti‐MOF@TpTt hybrids is verified by PXRD results. As shown in **Figure** **2**a, the peaks of all the Ti‐MOF@TpTt hybrids are similar to those of pure Ti‐MOF, indicating the surface graft of TpTt‐COF does not change the crystalline structure of Ti‐MOF. However, the characteristic peaks of TpTt‐COF cannot be clearly observed in the PXRD patterns of **1** and **2**, owing to the relatively weak crystallinity and low coating content of TpTt‐COF. With more coating, the appearance of a new characteristic diffraction peak at 27.4° in **3** and **4**, originating from the (002) plane of TpTt‐COF, confirmed the formation of TpTt‐COF in the Ti‐MOF@TpTt hybrids. Compared with the FTIR results of Ti‐MOF‐CHO, the characteristic peak of C═O stretching vibration in aldehyde groups at 1704 cm^–1^ disappeared in all the Ti‐MOF@TpTt hybrids, indicating the successful modification of TpTt shell on Ti‐MOF core through Schiff base reaction. Unlike the previous studies on MOF@COF materials with imine‐linked bonds,^[^ ^17^ ^]^ the stretching vibration of C═N at around 1620 cm^–1^ shows no significant changes on account of the *β*‐ketoenamine links in TpTt‐COF. Moreover, red‐shifts of C‐N stretching vibration from 1255 cm^–1^ at Ti‐MOF‐CHO to 1225 cm^–1^ at **4** provide further evidence for the covalent connection between Ti‐MOF core and TpTt shell. In addition, with an increased amount of TpTt‐COF precursors, a typical peak assigned to the stretching vibrations of triazine ring units at 805 cm^–1^ was observed and the peak's intensity gradually increases, whereas the intensity of peaks at 400–750 cm^–1^ corresponding to the Ti‐O‐Ti vibrations obviously decreases. Thus, the FTIR results demonstrate the Ti‐MOF@TpTt hybrids with a core–shell structure were successfully synthesized through covalent connection.

The XPS technique was also executed to explore the surface chemical composition and electronic state of elements in Ti‐MOF, **2**, and TpTt‐COF. As shown in Figure 2c, the N 1s XPS spectrum of **2** includes two peaks well corresponding to –N═C (398.7 eV) and –N‐C (399.98 eV), which show a slight positive shift as compared to that of TpTt‐COF. Interestingly, the peaks of –NH_2_ (399.38 eV) and –N^+^– (402.82 eV) belonging to amino groups in Ti‐MOF disappear in the N 1s region of **2**, revealing the full conversion of amine groups in Ti‐MOF and the successful formation of TpTt shell by covalent connection. In addition, the intensity of Ti‐O peak (530.33 eV) in O 1s spectrum of **2** obviously decreases as compared to that of Ti‐MOF, due to the shielding by the TpTt shell (Figure S7, Supporting Information). This result further confirms the successful synthesis of 2 with a core–shell structure, as the XPS is mainly used for the surface analysis, which is also in good agreement with the aforementioned FTIR analysis.

The permanent porosities of Ti‐MOF, TpTt‐COF, and Ti‐MOF@TpTt hybrids were evaluated by the N_2_ adsorption‐desorption experiments at 77 K (Figure 2d). The results indicated that all of the samples exhibited type I isotherm, which is a characteristic of the microporous material. The Brunauer‐Emmett‐Teller (BET) surface areas of Ti‐MOF and TpTt‐COF were determined to be 1385.1 and 166.43 m^2^ g^–1^, respectively. The BET values of **1** and **2** were calculated to be 1137.4 and 757.86 m^2^ g^–1^, which is much higher than that of TpTt‐COF. Their high BET surface areas can contribute to the exposure of active sites and rapid migration of reaction substrates. Whereas, **3** and **4** show quite low BET surface areas due to the high content of TpTt‐COF. The solid‐state ^13^C CP/MAS NMR spectrum of **2** shows all the characteristic peaks of TpTt‐COF and Ti‐MOF, which further confirms the successful growth of the TpTt shell and the structural integrity of the Ti‐MOF core during the hybridization process (Figure S8, Supporting Information). Moreover, the thermogravimetric analysis (TGA) proved that Ti‐MOF@TpTt hybrids have high thermal stability up to 360 °C (Figure S9, Supporting Information).

**Figure 3:**
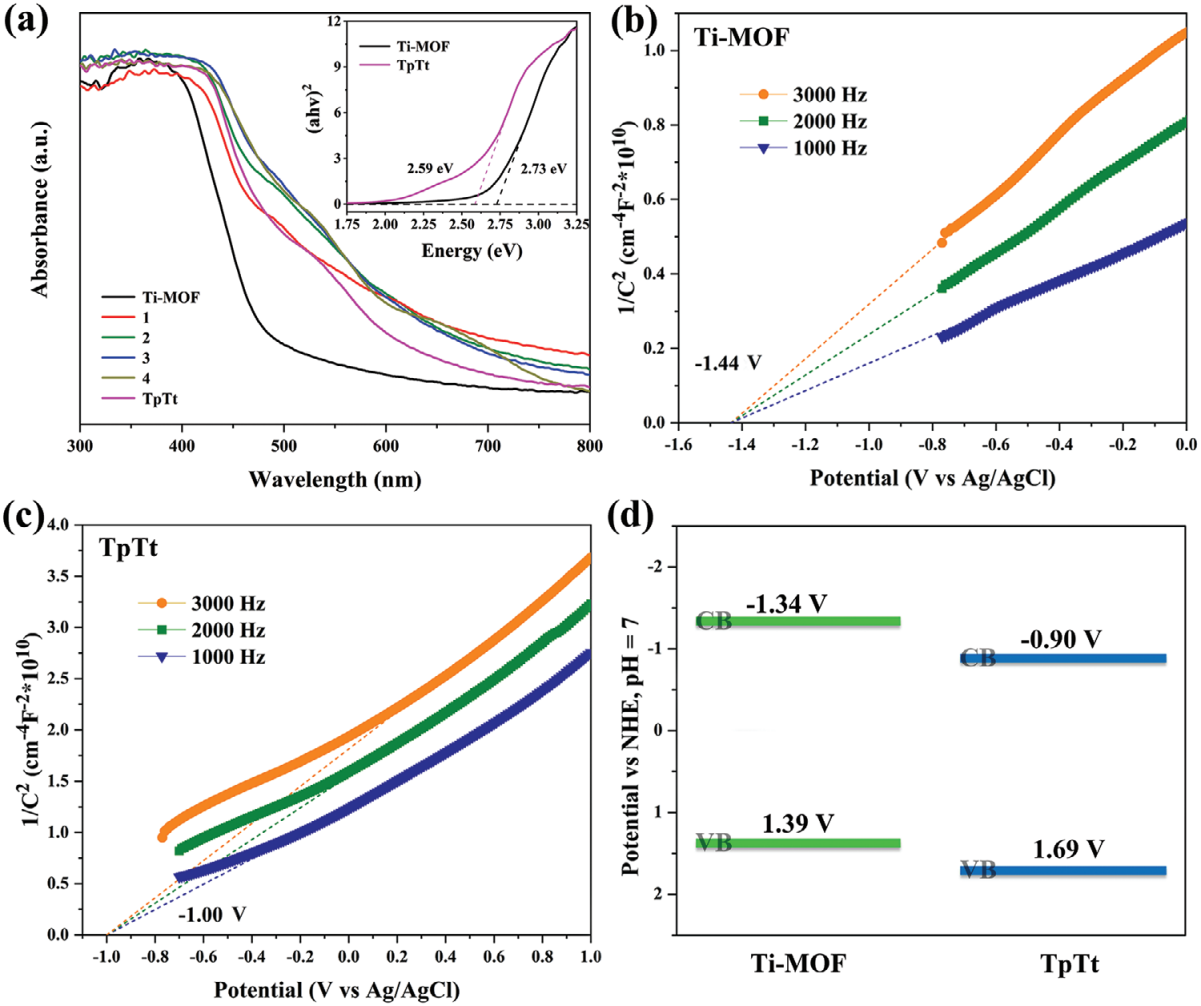
a) Solid‐state UV–Vis spectra of Ti‐MOF, TpTt‐COF, and Ti‐MOF@TpTt hybrids. The inset is the (*ahv*)^2^ versus energy plots of Ti‐MOF and TpTt‐COF; b) Mott‐Schottky plots of Ti‐MOF under different frequencies; c) Mott‐Schottky plots of TpTt‐COF under different frequencies; (d) Energy band structures of Ti‐MOF and TpTt‐COF.

The solid‐state UV–vis measurements were carried out to study the light‐harvesting properties of the Ti‐MOF, TpTt‐COF, and Ti‐MOF@TpTt hybrids. As shown in **Figure** **3**a, the absorption edges of Ti‐MOF and TpTt‐COF are 520 and 560 nm, respectively. As compared to Ti‐MOF, all the Ti‐MOF@TpTt hybrids exhibited obvious redshifts with broad absorption tail covering the entire visible range, implying their stronger visible light absorption performance. **2**–**4** show larger absorption edges than pure TpTt‐COF, demonstrating the synergistic effect between Ti‐MOF and TpTt‐COF. When the TpTt shell is too thin, the synergistic effect disappears and the absorption edge is similar to that of TpTt‐COF. The bandgap is calculated according to the Tauc plots as 2.73 eV for Ti‐MOF and 2.59 eV for TpTt‐COF. To estimate the flat band position (*V*_fb_) of Ti‐MOF and TpTt‐COF, Mott‐Schottky (MS) analysis was performed under three different frequencies. As presented in Figure 3b,c, both Ti‐MOF and TpTt show positive slopes, which is a characteristic of n‐type semiconductors. From the MS measurements, it can be deduced that the *V*_fb_ of Ti‐MOF and TpTt are −1.44 and −1.00 V versus Ag/AgCl (−1.24 and −0.80 V vs NHE), respectively. Generally, the bottom of the conduction band (CB) is more negative by about 0.1 V than the *V*_fb_ in n‐type semiconductors.^[^ ^13c^ ^]^ Thus, the CBs of Ti‐MOF and TpTt‐COF were calculated to be −1.34 and −0.90 V versus NHE, respectively. Combined with the bandgap energy, the valence band (VB) positions of Ti‐MOF and TpTt‐COF are 1.39 and 1.69 V versus NHE, respectively (Figure 3d). Therefore, upon hybridization, the photo‐generated electrons prefer to transfer from the Ti‐MOF core to the TpTt shell, leading to the spatial separation of photo‐generated electrons and holes, which can effectively improve the separation efficiency of photo‐generated electrons and holes. Thus, **2** was chosen as a promising platform for the following photocatalytic reactions due to its high BET surface area and strong absorption ability for visible light.

**Figure 4:**
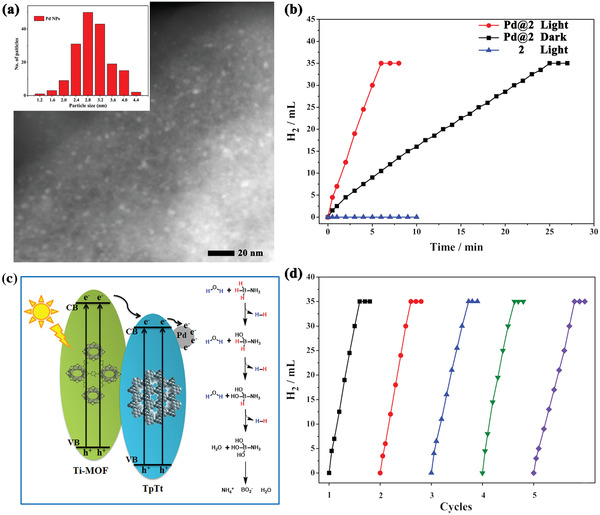
a) The enlarged HADDF‐STEM images of Pd@**2**. Inset: size distribution of Pd NPs; b) Plots of time versus volume of hydrogen generation from AB aqueous solution over Pd@**2** and **2** under different conditions; c) Proposed mechanism for the AB hydrolysis catalyzed by Pd@**2**; d) Cycling of AB hydrolysis under light irradiation over Pd@**2**.

As we all know, Pd nanoparticles (NPs) show excellent catalytic behavior in various reactions due to the unique chemical and physical properties.^[^ ^18^ ^]^ Therefore, in this work, Pd NPs were decorated on the TpTt shell as electron traps to concentrate the photo‐generated electrons and facilitate the photo‐generated electrons flow from the Ti‐MOF core to the TpTt shell. The as‐prepared Pd^2+^@**2** precursor can be in situ reduced during the following photocatalytic cascade reactions to afford the final photocatalyst Pd@**2**. Homogeneously distributed Pd NPs show an average size of 2.8 ± 0.37 nm as confirmed by the HADDF‐STEM and EDX‐mapping images (**Figure** **4**a and Figure S10, Supporting Information). The structural integrity of Pd@**2** maintains well during the Pd loading process, according to the well‐matched PXRD patterns and similar BET surfaces with **2** (Figures S11 and S12, Supporting Information). Meanwhile, no identifiable diffraction peaks of Pd NPs were observed in Pd@**2** from the PXRD profile, implying the low Pd content and/or the small size of Pd NPs in Pd@**2**. The actual content of Pd in Pd@**2** is detected to be 2.95% by Inductively Coupled Plasma Optical Emission Spectrometer (ICP‐OES). Pd@**2** shows a similar light absorption ability to **2**, which indicates that loading Pd NPs does not influence the bandgap of **2** (Figure S13, Supporting Information).

It is well‐known that ammonia borane (AB, NH_3_BH_3_) is a leading contender for chemical hydrogen storage mainly due to its high hydrogen content of 19.6 wt%.^[^ ^19^ ^]^ Besides, by virtue of its remarkable stability and high solubility in solution under ambient conditions, AB is an excellent transfer hydrogenation agent for various hydrogenation reactions.^[^ ^20^ ^]^ Prior to the cascade hydrogenation reaction with AB as the hydrogen resource, the AB hydrolysis performance was investigated under light irradiation and in the dark. To eliminate the thermal influence, the reaction temperature in the photocatalytic process was kept at 298 K by circulating cool water. As shown in Figure 4b, completed hydrolysis of AB with an H_2_/AB ratio of 3.0 can be achieved in 25 min over Pd@**2** in the dark. Remarkably, the reaction can be completed in just 6 min under light irradiation, affording a TOF value of 180.37 mol H_2_ mol_Pd_^–1^ min^–1^. The results indicate that the catalytic performance of AB hydrolysis is significantly enhanced by light irradiation presumably because of the formation of electron‐rich Pd sites on Pd@**2** under light irradiation. However, no reaction occurs with **2** under light irradiation, implying that Pd NPs are the active sites. Furthermore, the AB hydrolysis performance of Pd@**1**, Pd@**3**, Pd@**4**, Pd@Ti‐MOF, and Pd@TpTt were also investigated under the same catalytic conditions. As shown in Figure S14 (Supporting Information), Pd@Ti‐MOF and Pd@TpTt completely finished the reaction under light irradiation within 10 and 11 min, respectively, which is due to the good light‐harvesting ability of Ti‐MOF and TpTt‐COF. Interestingly, Pd@**2** and Pd@**3** exhibit better catalytic performance than Pd@Ti‐MOF and Pd@TpTt. The enhanced activity can be attributed to the core–shell structure and type II heterojunction between Ti‐MOF and TpTt‐COF, which promotes the spatial separation of photo‐generated electrons and holes. However, Pd@**1** shows similar catalytic ability to that of Ti‐MOF and TpTt‐COF due to the ultrathin TpTt shell and the disappearance of the heterojunction. Pd@**4** shows the lowest catalytic performance among all the catalysts, owing to the shielding effect of the ultrathick TpTt shell. Thus, we can conclude that the core–shell structure coated with ultrathin nanobelt TpTt shell and the type II heterojunction between Ti‐MOF and TpTt‐COF are the key to the enhanced activity of Ti‐MOF@TpTt hybrids.

To investigate the mechanism for AB hydrolysis over Pd@**2** under light irradiation, FTIR, GC, ^11^B NMR, and kinetic isotopic experiments were further tested. The FTIR results of before, during, and after AB hydrolysis demonstrate the formation of _3_‐BH*_x_* (OH)_3−_ *_x_* and ammonium borate hydrates (Figure S15, Supporting Information).^[^ ^15^, ^21^ ^]^ According to the results of GC and ^11^B NMR, the H_2_ and BO_2_^–^ are the final products after AB hydrolysis over Pd@**2** (Figures S16 and S17, Supporting Information). The kinetic isotopic experiment of AB hydrolysis over Pd@**2** was carried out upon changing the reactant from H_2_O to D_2_O (Figure S18, Supporting Information). As compared to H_2_O as the reactant, Pd@**2** shows much slower H_2_ evolution rate when using D_2_O as the reactant, indicating that the activation of the water molecule or the cleavage of the O—H bond is the rate‐determining step for AB hydrolysis. ^[^ ^22^ ^]^ Based on the above results, a mechanism for the photo‐enhanced catalytic performance of AB hydrolysis over Pd@**2** is proposed. When the core–shell hybrid is exposed to light, photo‐generated electrons not only migrate from the Ti‐MOF core to the TpTt shell but also directly generate on the TpTt shell, and then concentrate on the highly dispersed Pd NPs. The electron‐rich Pd NPs with the plasmonic surface favor enhanced binding of AB and the activation of water molecules, which effectively weaken the B—H bond in AB and H—O bond in water molecules, thus resulting in the superior hydrogen evolution performance. Moreover, the recyclability of Pd@**2** was also performed, and no notable decrease in activity after 5 cycles was observed, suggesting the excellent stability of Pd@**2** (Figure 4d). The PXRD patterns of Pd@**2** before and after AB hydrolysis showed no obvious change, further confirming that Pd@**2** has good stability (Figure S19, Supporting Information). The HAADF‐STEM image confirms that the Pd NPs did not display obvious aggregation after the reactions, and the morphology was well maintained (Figure S20, Supporting Information).

The catalytic activity of Pd@**2** for the one‐pot cascade reactions involving the hydrolysis of AB and nitrobenzene hydrogenation at 298 K was also investigated. The results are shown in **Tables** **1** and **2**. Pd@**2** exhibits decent catalytic performance and completes the conversion with >99% selectivity within 10 min in pure water under light irradiation. The hydrogenation of nitrobenzene did not occur without AB, implying the importance of H_2_ resource. Nitrobenzene was unable to completely convert to aniline within 10 min in the dark, mainly owing to the low H_2_ evolution rate from AB hydrolysis in the dark, which indicates that the active hydrogen originated from the AB hydrolysis plays a significant role in the hydrogenation of nitrobenzene. Although the methanolysis rate of AB commonly slows than that of AB hydrolysis, methanol is always used to improve the dissolution of various nitroarenes.^[^ ^23^ ^]^ The result of an incomplete conversion of nitrobenzene in the mixture of methanol and H_2_O within 10 min further implied the importance of the active hydrogen originated from the AB hydrolysis. To confirm our hypothesis, gaseous H_2_ was used to replace AB as the H_2_ resource. The conversion of nitrobenzene was only about 5% within 10 min when using H_2_ (1 atm) instead of AB. Thus, the above results demonstrate that the active hydrogen originated from the AB hydrolysis is much more efficient than the classical H_2_ resource for the hydrogenation of nitrobenzene. Moreover, no reaction product can be obtained in the absence of Pd NPs although the AB was introduced as the H_2_ resource, suggesting that Pd NPs are the active sites for the hydrogenation of nitrobenzene. In addition, no Pd element was detected by ICP‐OES analysis in the solution after the reaction, suggesting the absence of Pd leaching.

**Table 1:** One‐pot cascade hydrogenation of nitrobenzene over Pd@**2** with AB as the H_2_ resource

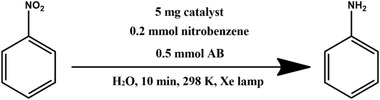					
Entry	Catalyst	Light	H_2_ Source	Conversion/%	Selectivity/%
1	Pd@**2**	√	AB	>99	>99
2^a)^	Pd@**2**	√	AB	92	>99
3	Pd@**2**	×	AB	92	>99
4	Pd@**2**	√	H_2_	5	>99
5	**2**	√	AB	Trace	Trace
6	Pd@**2**	√	×	Trace	Trace
7^b)^	Pd@**2**	√	AB	97	>99

^a)^ Reaction condition: 1 mL methanol and 2 mL H_2_O

^b)^ Reaction condition: In the 5th reaction run.

**Table 2:** One‐pot cascade hydrogenation of nitroarenes over Pd@**2**

Entry	Substrate	Product	Conversion/%	Selectivity/%
1	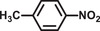	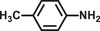	94	>99
2	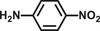	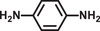	>99	>99
3	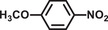	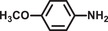	98	>99
4	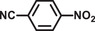	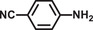	93	>99
5	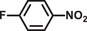	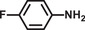	>99	>99
6	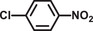	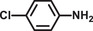	96	>99
7	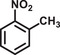	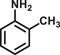	93	>99
8	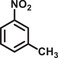	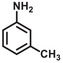	94	>99
9	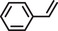	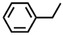	>99	>99

Reaction conditions: 5 mg Pd@**2**, 0.2 mmol nitroarenes, 0.5 mmol AB, 1 mL methanol, and 2 mL H_2_O at 298 K under light irradiation.

**Figure 5:**
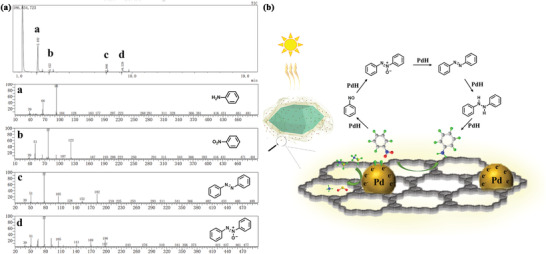
a) GC‐MS of the production in the reaction of nitrobenzene hydrogenation after reacting for 3 min, a–d are GC‐MS of aniline, nitrobenzene, azobenzene, and azoxybenzene, respectively; b) The mechanism of nitrobenzene hydrogenation under light irradiation over Pd@**2**.

To understand the reaction mechanism of nitrobenzene hydrogenation with AB as the H_2_ resource, the photocatalyst was filtered out after reacting for 3 min. The filtrate was analyzed by GC‐MS as shown in **Figure** **5**a. Azobenzene and azoxybenzene were captured in the filtrate, which can be considered as intermediate products. Thus, the reaction pathway of nitrobenzene hydrogenation was proposed according to the intermediate products, as shown in Figure 5b. More significantly, the hydrogenation performance of various nitroarene derivatives over Pd@**2** was also investigated as shown in Table 2. Regardless of the type or position of the substituents, Pd@2 showed superior photocatalytic performance toward the hydrogenation of various nitroarenes. Besides, Pd@**2** also catalyzed styrene to ethylbenzene with high conversion (>99%) and selectivity (>99%) within 10 min under light irradiation.

To test the recyclability and stability of Pd@2, the used photocatalyst was separated, washed, and dried after each reaction. As shown in Figure S21 (Supporting Information), the photocatalyst still showed high catalytic activity and selectivity with negligible changes after 5 cycles under the same conditions, implying the superior stability of Pd@**2** (Figure S22, Supporting Information).

## 3 Conclusion

In summary, novel core–shell Ti‐MOF@TpTt hybrids with controllable morphology adjustment of COF shell were synthesized through a sequential growth strategy. By rationally controlling the ratio of Ti‐MOF‐CHO and the TpTt‐COF precursors, the Ti‐MOF@TpTt hybrid coated with ultra‐thin COF nanobelt‐shell, integrating high BET surface area and strong light absorption ability, was successfully achieved. The striking core–shell feature, strong light absorption ability, as well as well‐matched bandgap, and the strong covalent connection between Ti‐MOF and TpTt‐COF, significantly promote the separation efficiency of photo‐generated electrons and holes. Moreover, the high BET surface and ultra‐thin COF nanobelt shell offer more accessible reactive sites and reduce the migration distance for the substrates, which endow Ti‐MOF@TpTt composite a perfect platform for photocatalytic reactions. Thus, the as‐obtained Pd decorated Pd@Ti‐MOF@TpTt catalyst exhibited much higher photocatalytic performance than Ti‐MOF, TpTt‐COF, and the hybrids with fibrillar‐like COF‐shell for the cascade reactions of AB hydrolysis and nitroarenes hydrogenation. Finally, the possible mechanisms of AB hydrolysis and nitroarenes hydrogenation were also proposed to well explain the improved performance of this photocatalytic system. This work provides an effective strategy to adjust the morphology of COF shell in the synthetic process of MOF@COF hybrids and explores the reasons for the improvement of photocatalytic performance due to the morphology adjustment, which will expand MOF/COF hybrids and broaden their applications in photocatalysis.

## 4 Experimental Section

### Chemicals

2‐Aminoterephthalic acid (H_2_BDC‐NH_2_, Sigma Aldrich Co.), Titanium (IV) *n*‐butoxide (Ti(OC_4_H_9_)_4_, TCI Co.), 1,3,5‐triformylphloroglucinol (Tp, EXTENSION Co.), Melamine (Tt, Energy Chemical Co.), Ammonia borane (AB, Aladdin), Palladium acetate (Aladdin). All chemicals are analytical grade and used as received without further purification.

### Synthesis of MIL‐125‐NH_2_(Ti) (Ti‐MOF)

Typically, H_2_BDC‐NH_2_ (2.7 g, 15 mmol) and (Ti(OC_4_H_9_)_4_) (1.3 mL, 3.75 mmol) were dissolved to a mixture of dry *N,N*‐dimethylformamide (DMF, 45 mL) and dry methanol (5 mL), followed by stirring at room temperature for ≈30 min. The above mixture was transferred into a 100 mL Teflon‐lined stainless‐steel autoclave and heated at 150 °C for 72 h. After cooling to room temperature, the yellow precipitate was washed by DMF and methanol several times, followed by drying at 80 °C under vacuum for 12 h.

### Synthesis of TpTt‐COF

A *N,N*‐dimethylacetamide (DMAc)/dimethyl sulfoxide (DMSO) (2/1 mL) mixture of Tp (0.3 mmol, 63 mg) and Tt (0.3 mmol, 38 mg) in the presence of an acetic‐acid catalyst (6 m, 0.3 mL) in a Pyrex tube (10 mL) was degassed through three freeze‐pump‐thaw cycles. The tube was flame sealed and heated at 120 °C for three days. After cooling to room temperature, the precipitate was isolated by centrifugation and washed with a copious amount of DMAc and acetone. Then the powder material was dried at 80 °C under vacuum for 12 h to get the as‐synthesized TpTt‐COF.

### Synthesis of Ti‐MOF@Tp (Ti‐MOF‐CHO)

30 mg Ti‐MOF and 15 mg Tp were added into a Schlenk tube containing 2 mL DMAc and 1 mL DMSO, and the suspension was sonicated for 15 min. After adding 0.1 mL HAc (6 m) to the suspension, it was heated at 80 °C for 24 h. Subsequently, the precipitate was centrifuged, washed with DMAc and acetone, and dried at 80 °C under vacuum for 12 h to get the as‐synthesized Ti‐MOF‐CHO.

### Synthesis of Ti‐MOF@TpTt‐1 (1)

30 mg Ti‐MOF‐CHO, 10 mg Tp, and 5 mg Tt were added to a mixture of 2 mL DMAc and 1 mL DMSO into a 10 mL Pyrex tube. After the mixture was sonicated for 15 min, 6 mHAc (0.1 mL) was added. Then, the Pyrex tube was degassed through three freeze‐pump‐thaw cycles, sealed off, and heated at 120 °C for 72 h. The resulting precipitate was isolated by centrifugation, washed with DMAc and acetone, and dried at 80 °C under vacuum for further use.

### Synthesis of Ti‐MOF@TpTt‐2 (2)

The synthesis of **2** is similar to that of 1 but using 15 mg Tp and 10 mg Tt.

### Synthesis of Ti‐MOF@TpTt‐3 (3)

The synthesis of **3** is similar to that of 1 but using 30 mg Tp and 20 mg Tt.

### Synthesis of Ti‐MOF@TpTt‐4 (4)

The synthesis of **4** is similar to that of 1 but using 63 mg Tp and 38 mg Tt.

### Synthesis of Pd^2+^/Ti‐MOF@TpTt

3.2 mg of palladium acetate was dissolved in 2 mL of dichloromethane (DCM) and then 30 mg of Ti‐MOF@TpTt was added. The suspension was stirred at room temperature for 24 h. The suspension was isolated by centrifugation, washed with DCM, and then dried at 70 °C to get the Pd^2+^/Ti‐MOF@TpTt. Pd^0^/Ti‐MOF@TpTt was in situ synthesized in the photocatalytic reactions.

### Characterizations

Powder X‐ray diffraction (PXRD) was recorded on a Rigaku MiniFlex 600 diffractometer using Cu K*α* radiation (*λ* = 1.5418 Å) at 30 kV. Fourier transform infrared spectra (FTIR) were analyzed on a Bruker ALPHA FTIR spectrometer from KBr pellets as the sample matrix. The solid‐state UV–vis absorption spectra were performed by Agilent Cary 5000 UV‐VIS‐NIR spectrometer, and standard barium sulfate was used as the reference material. The X‐ray photoelectron spectroscopy (XPS) measurements were performed with a VG Scientific ESCALAB 250 instrument with an Al K*α* (300 W) X‐ray resource. The nitrogen sorption isotherms were measured with a Belsorp Max automatic volumetric adsorption system at liquid nitrogen temperature (77 K) using N_2_ as the probe gas after a degassing process at 100 °C for 12 h under vacuum. The specific surface areas were obtained by using the Brunauer–Emmett–Teller (BET) model. Scanning electron microscopy (SEM) was performed to investigate the particle morphology using a Zeiss Sigma 500 scanning electron microscope at an accelerating voltage of 1.5–3.0 kV. ^13^C MAS solid‐state NMR experiments were performed on Agilent 600 DD2 spectrometer at a resonance frequency of 150.15 MHz. Thermogravimetric analysis (TGA) was recorded by a TGA Q50 thermal analyzer from room temperature to 800 °C under N_2_ atmosphere using a heating rate of 10 °C min^−1^. Inductively coupled plasma optical emission spectrometer (ICP‐OES) was recorded with SHIMADZU ICPE‐9820. Transmission electron microscopy (TEM) was performed with a FEI TalosF200s high‐resolution transmission electron microscope with an accelerating voltage of 200 kV. EDS system: 2SDD windowless design, shutter‐protected. Fast EDS mapping: pixel dwell times down to 10 µs. ^11^B NMR spectra were recorded using Bruker AVIII HD 600 MHz NMR spectrometer. Py‐GC/MS was measured on a CDS5200HP‐R QP2010 Ultra spectrometer.

### The FTIR Spectra of Before, During, and After AB hydrolysis

The reaction solution (10 µL) before, during, and after AB hydrolysis was dropped on the freshly prepared KBr pellet, respectively. The FTIR spectra of before, during, and after AB hydrolysis were measured after drying the KBr pellets. The reaction solution before AB hydrolysis was prepared by dissolving 0.5 mmol AB in 3 mL water. The reaction solution during and after AB hydrolysis was collected when the photocatalytic reaction of AB hydrolysis proceeded to 3 min and more than 30 min, respectively.

### The Mott‐Schottky Measurement

The Mott‐Schottky (MS) analysis was recorded on the CHI660E electrochemical workstation (Chenhua Instrument, Inc.) in a standard three‐electrode system with the photocatalyst‐coated ITO as the working electrode, a Pt wire as the counter electrode, Ag/AgCl as a reference electrode, and aqueous Na_2_SO_4_ (0.5 mol L^−1^) solution as the electrolyte. Finally, the Mott‐Schottky (MS) plots were performed under 1000, 2000, and 3000 Hz.

### Hydrolysis Reaction of AB

The AB hydrolysis was conducted in water at 298 K under light irradiation and a gas collecting device was connected to the reaction flask to measure the volume of released gas (Figure S23, Supporting Information). In general, 5 mg photocatalyst and 2 mL DI water were placed into a vial. The reaction started when the freshly prepared AB solution (0.5 M, 1 mL) was injected into the above vial. The reaction was performed under the irradiation of a 300 W Xe lamp. The generation of H_2_ was confirmed by using an Agilent GC7820 Gas Chromatograph. For the recycling test, the photocatalyst was filtered after the reaction, dried, and was reused in the subsequent reaction under identical reaction conditions.

### Cascade Dehydrogenation of Various Nitroarenes Coupling with AB Hydrolysis

Typically, a mixture of 0.2 mmol of nitrobenzene and 5 mg photocatalyst was dispersed in a 25 mL Schlenk tube with 2 mL DI water. The reaction was carried out with the irradiation of a 300 W Xe lamp. The reaction got started when 1 mL of 0.5 M AB aqueous solution was added into the Schlenk tube. The reaction products were analyzed by Gas Chromatography (Agilent 6890N). For the recycling test, the photocatalyst was filtered after the reaction, dried, and was reused in the subsequent reaction under identical reaction conditions.

## Conflict of Interest

The authors declare no conflict of interest.

## Supporting information

Supporting Information

## Data Availability

The data that supports the findings of this study are available in the supplementary material of this article.
