# Supplementary material for: Construction of Core–Shell MOF@COF Hybrids with Controllable Morphology Adjustment of COF Shell as a Novel Platform for Photocatalytic Cascade Reactions
Source: Adv Sci (Weinh). 2021 Aug 10;8(19):2101884. doi: 10.1002/advs.202101884 (PMC8498909; doi:10.1002/advs.202101884)
Supplement: Supplementary file 1 — Supporting Information [file ADVS-8-2101884-s001.pdf]

WILEY-VCH

© 2021 Wiley-VCH GmbH

ADVANCED SCIENCE
Open Access

Supporting Information

for *Adv. Sci.,* DOI: 10.1002/advs.202101884

Construction of Core-shell MOF@COF Hybrids with Controllable Morphology Adjustment of COF Shell as a Novel Platform for Photocatalytic Cascade Reactions

Meng-Yao Zhang, Jun-Kang Li, Rui Wang, Shu-Na Zhao, Shuang-Quan Zang*, and Thomas C. W. Mak*

M. -Y. Zhang, J. -K. Li, Dr. R. Wang, Dr. S. -N. Zhao, Prof. S. -Q. Zang, Prof. T. C. W. Mak
Henan Key Laboratory of Crystalline Molecular Functional Materials
Henan International Joint Laboratory of Tumor Theranostical Cluster Materials
Green Catalysis Center and College of Chemistry
Zhengzhou University
Zhengzhou 450001, P. R. China
E-mail: zangsqzg@zzu.edu.cn

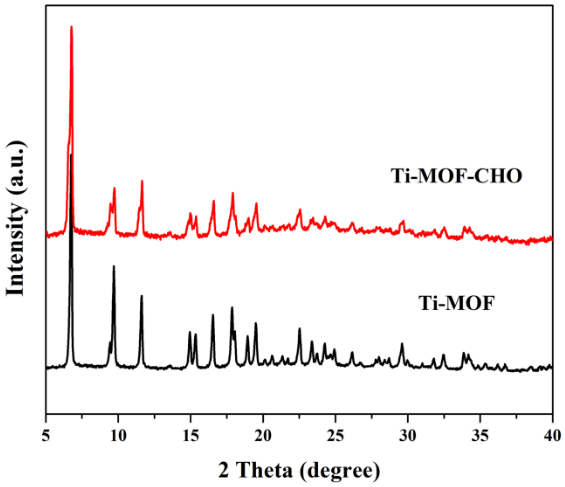

Figure S1. PXRD patterns of Ti-MOF and Ti-MOF-CHO.

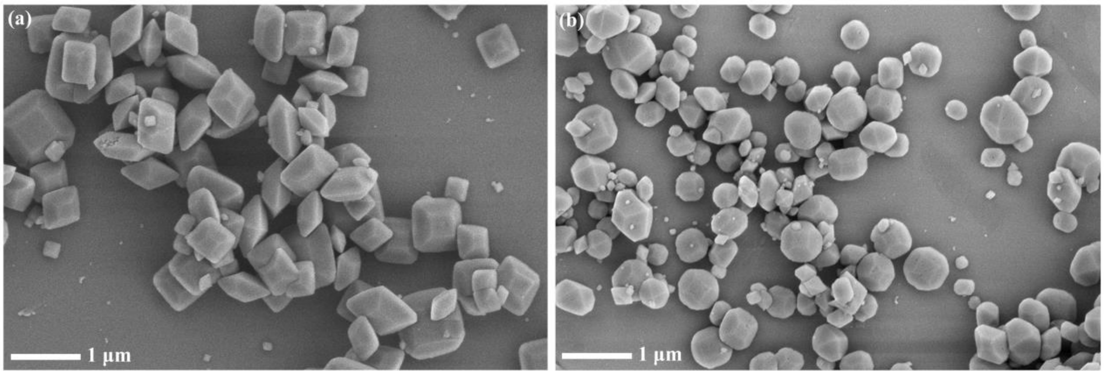

Figure S2. SEM images of (a) Ti-MOF and (b) Ti-MOF-CHO.

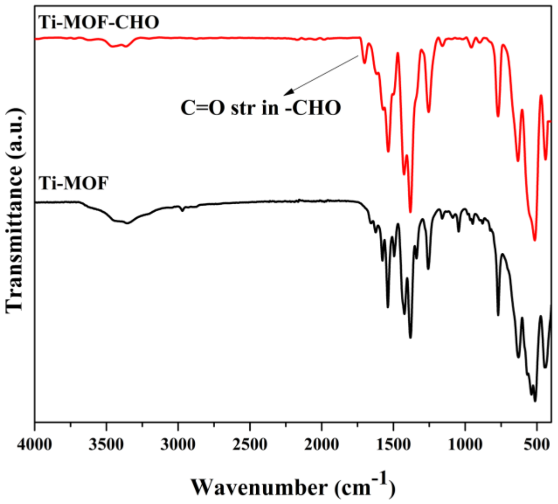

Figure S3. FTIR spectra of Ti-MOF and Ti-MOF-CHO.

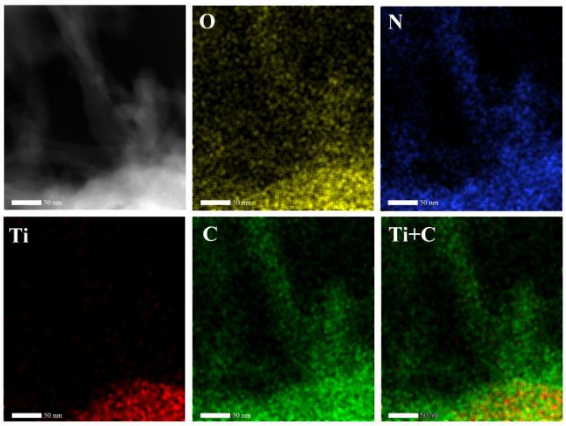

Figure S4. HAADF-STEM and elemental mapping images of **2**.

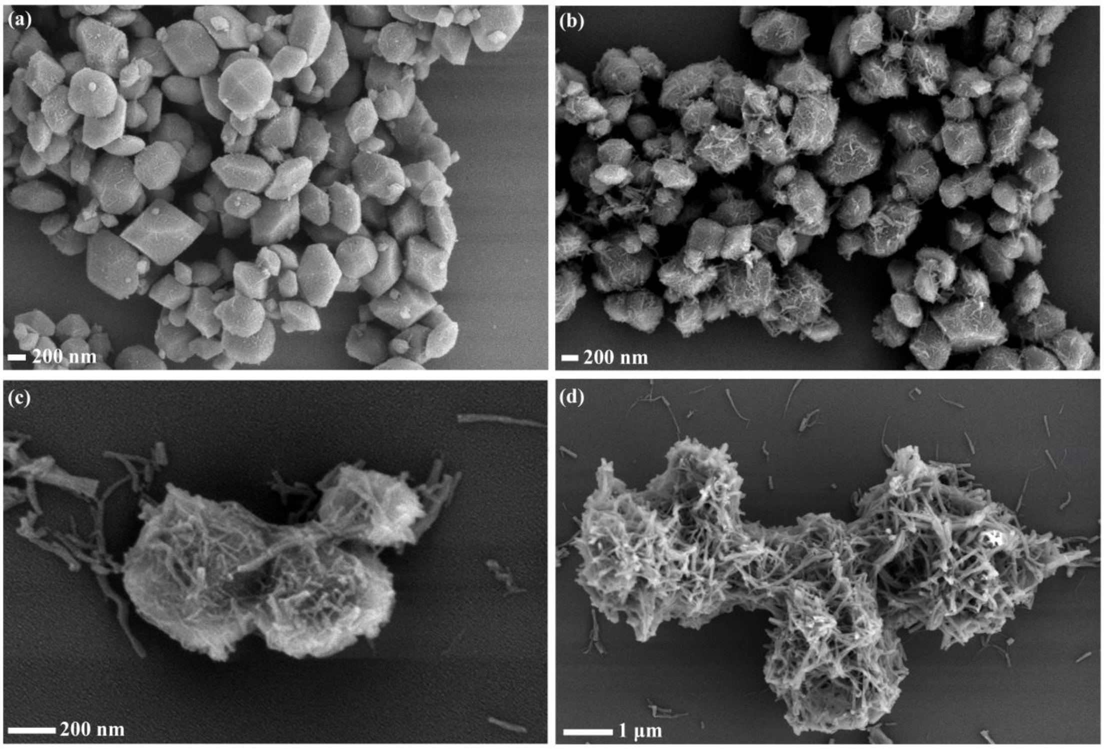

Figure S5. SEM images of Ti-MOF@TpTt hybrids with different amount of TpTt-COF precursors.

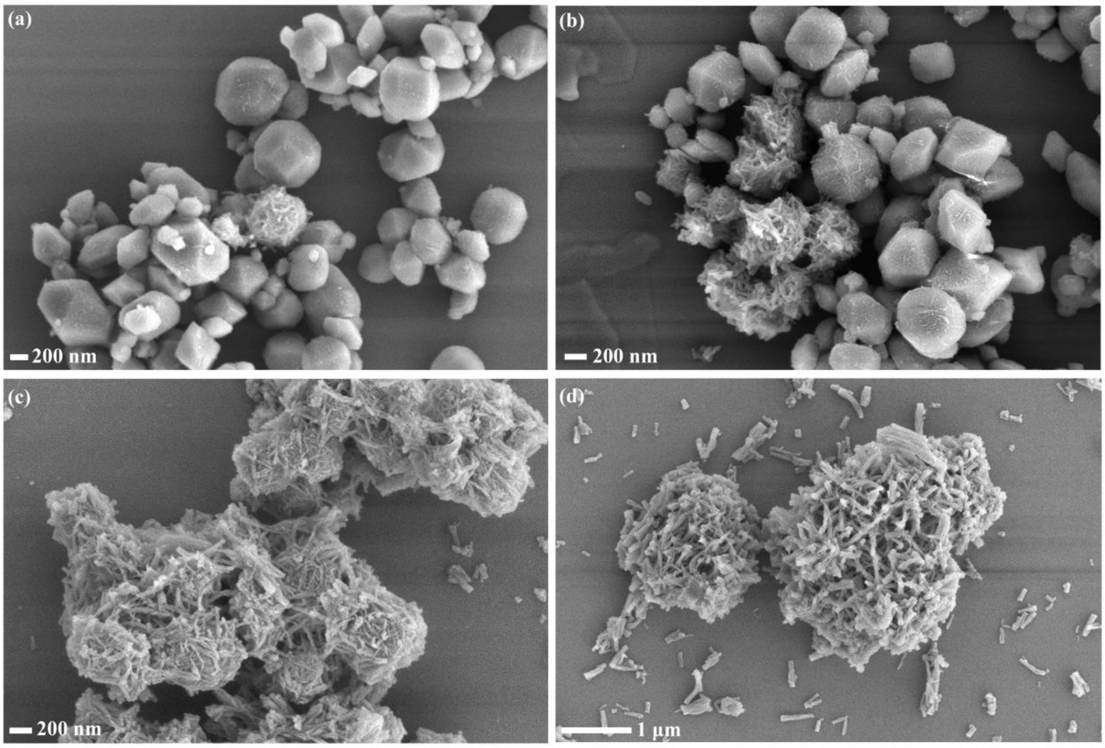

Figure S6. SEM images of Ti-MOF@TpTt hybrids without aldehyde modification for Ti-MOF.

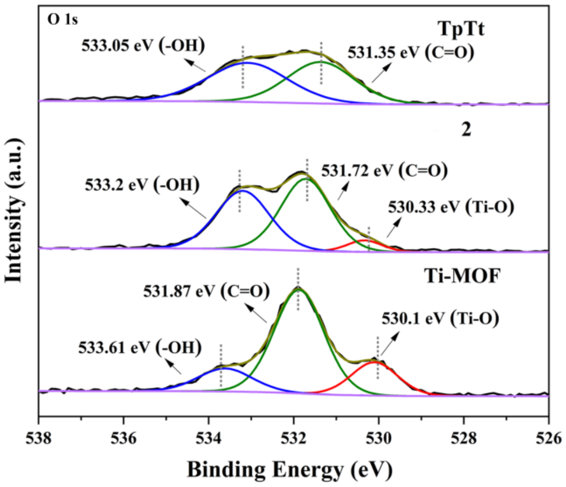

Figure S7. O 1s spectra of Ti-MOF, **2**, and TpTt-COF.

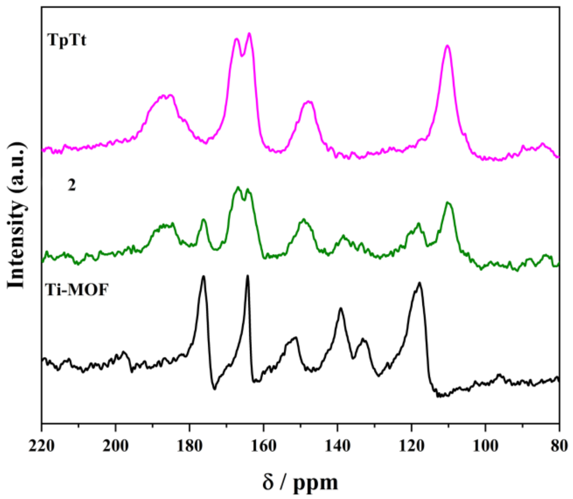

Figure S8. The solid-state ^{13}C CP/MAS NMR spectra of Ti-MOF, **2**, and TpTt-COF.

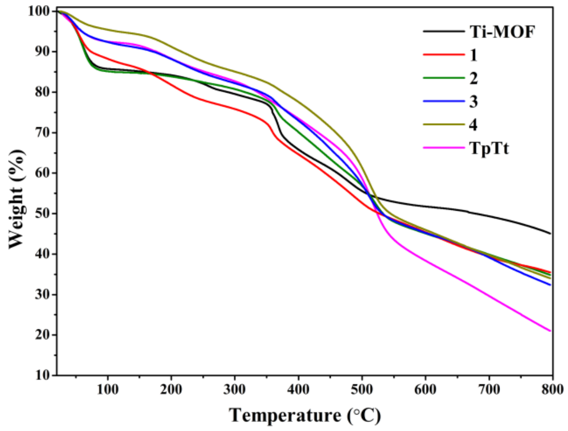

Figure S9. The TGA curves of Ti-MOF, Ti-MOF@TpTt hybrids, and TpTt-COF.

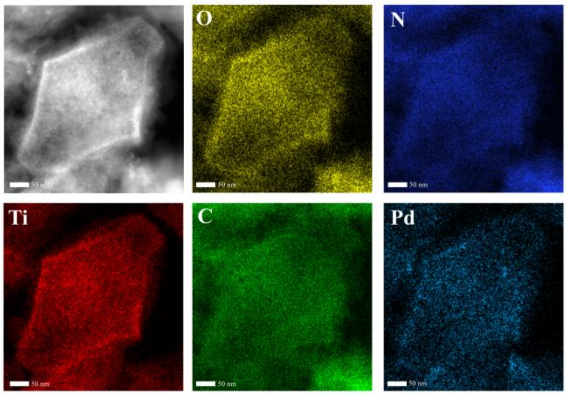

Figure S10. HAADF-STEM and elemental mapping images of Pd@**2**.

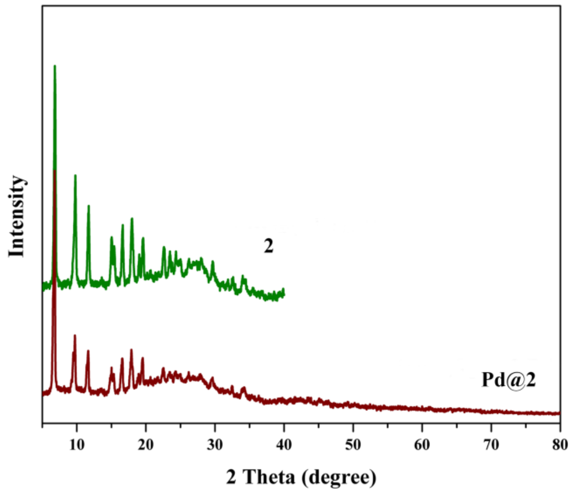

Figure S11. PXRD patterns of **2** and Pd@**2**.

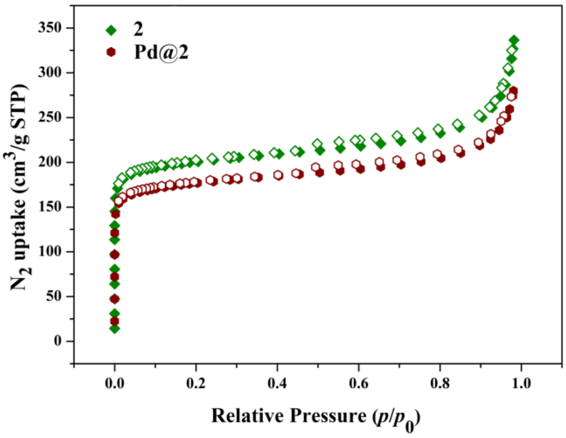

Figure S12. N_2 adsorption-desorption isotherms of **2** and Pd@**2**.

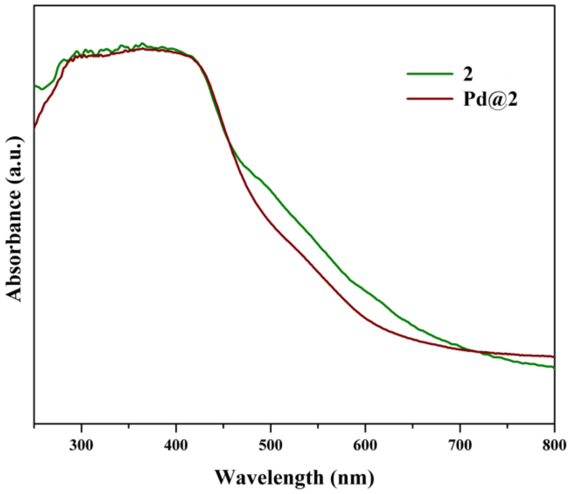

Figure S13. Solid-state UV-Vis absorption spectra of **2** and Pd@**2**.

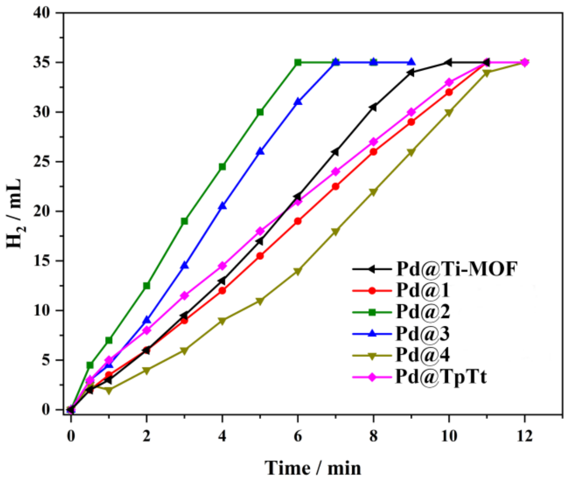

Figure S14. Catalytic activity of H_2 generation from AB hydrolysis over various photocatalysts under light irradiation.

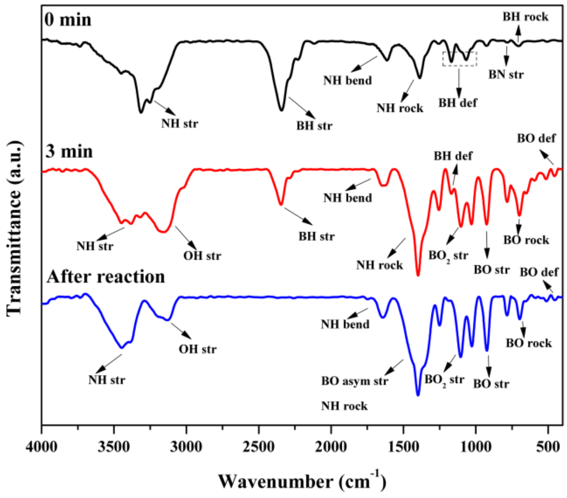

Figure S15. FTIR spectra of before, during, and after photocatalytic AB hydrolysis over Pd@**2**.

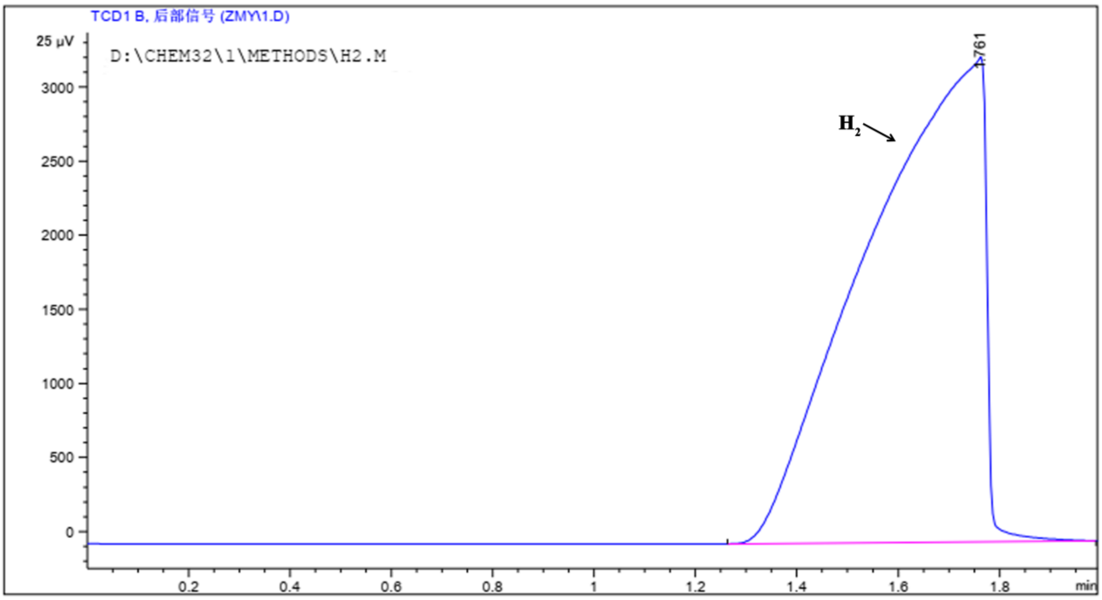

Figure S16. GC result for the released gas from AB hydrolysis over Pd@**2**.

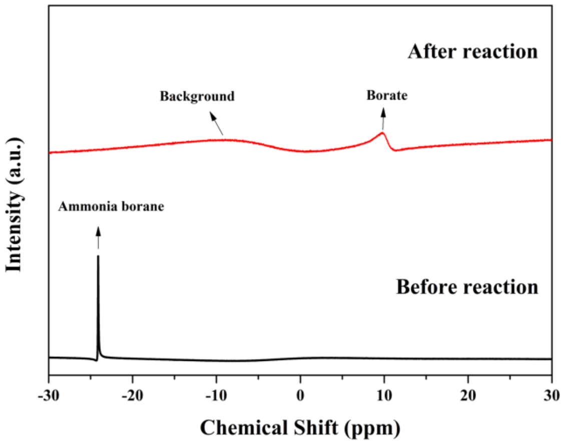

Figure S17. ^{11}B-proton decoupled NMR spectra of freshly prepared aqueous AB solution and reacton product after hydrolysis of AB.

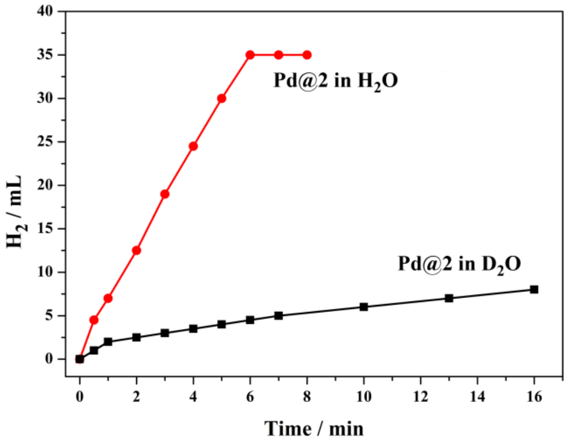

Figure S18. Kinetic isotope measurements and catalytic activity of H_2 generation from AB hydrolysis. The volume of the H_2 generated from AB (0.5 mmol) hydrolysis versus time using H_2O and D_2O as the reactants at 298 K over Pd@**2**.

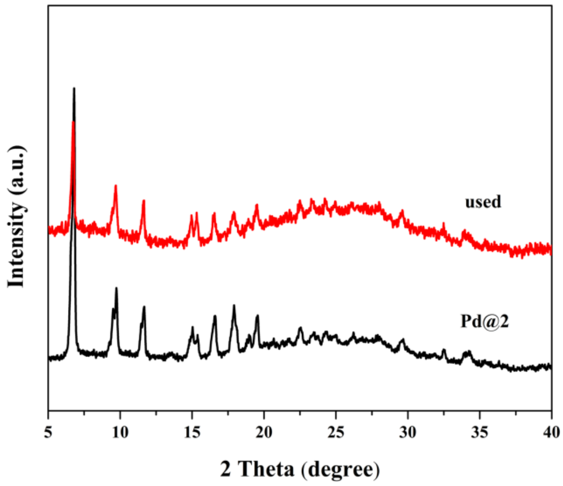

Figure S19. PXRD patterns of Pd@**2** before and after photocatalytic AB hydrolysis.

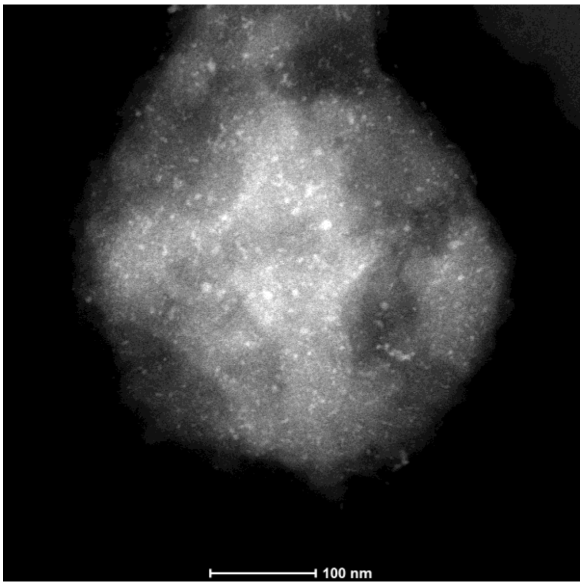

Figure S20. HAADF-STEM image of Pd@**2** after photocatalytic AB hydrolysis.

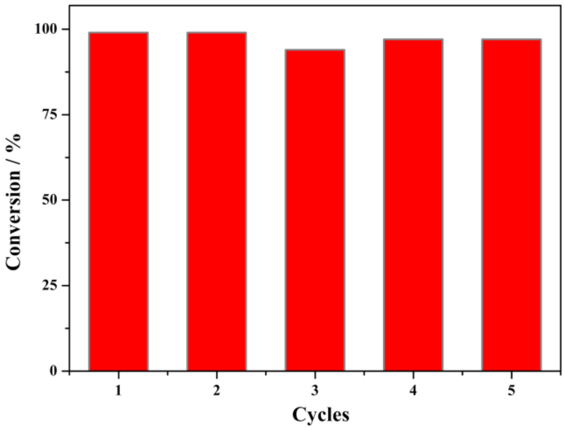

Figure S21. Cycling of nitrobenzene hydrogenation under light irradiation over Pd@**2**.

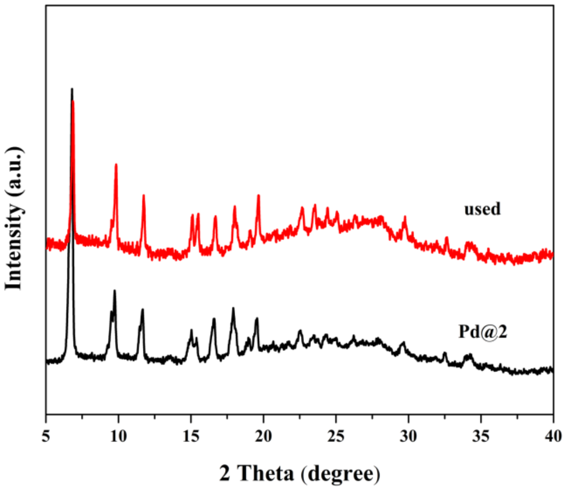

Figure S22. PXRD patterns of Pd@**2** before and after photocatalytic hydrogenation of nitrobenzene.

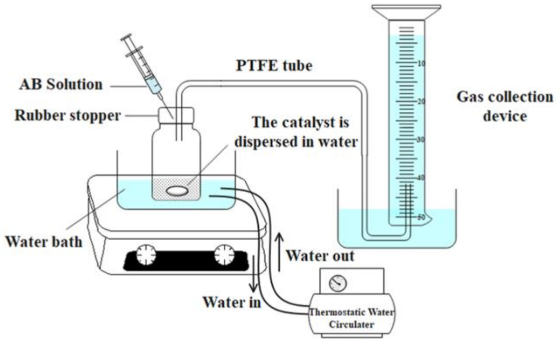

Figure S23. The experimental setup for AB hydrolysis.
